# Editorial: Novel insights into cellular mechanisms and therapies for tissue regeneration

**DOI:** 10.3389/fcell.2026.1796515

**Published:** 2026-02-10

**Authors:** Weiqiang Lan, Yuheng Liu, Zhangheng Huang, Chuan Guo

**Affiliations:** Department of Orthopedic Surgery and Orthopedic Research Institute, West China Hospital, Sichuan University, Chengdu, Sichuan, China

**Keywords:** extracellular vesicles, immunomodulation, microenvironment, stem cells, tissue regeneration

## Introduction

Tissue regeneration represents one of the most profound challenges in modern medicine, requiring a delicate orchestration of cellular behaviors, microenvironmental cues, and immunomodulatory signals. The shift from traditional tissue replacement to biological repair has been driven by a deeper understanding of how cells—both endogenous and transplanted—interact with their niche (Lane et al., 2014). This Research Topic, “Novel Insights into Cellular Mechanisms and Therapies for Tissue Regeneration,” curates a Research Topic of nine articles that explore the cutting edge of regenerative medicine. From the fundamental biology of stem cell niches and “super-regenerator” models to the translational potential of extracellular vesicles (EVs) and microenvironment-modulating strategies, these studies collectively highlight a paradigm shift towards precision regenerative therapies.

## The stem cell niche and intrinsic regenerative capacity

The foundation of tissue repair lies in the intrinsic potential of stem cells and their regulation by the local microenvironment, or niche. Understanding how specific niche components dictate stem cell fate is crucial for optimizing *in vitro* culture and *in vivo* transplantation. Aghazadeh et al. provide critical insights into this interaction by investigating Limbal Epithelial Stem Cells (LESCs), which are essential for corneal transparency. Their work reveals that fibronectin, a key extracellular *matrix* component, significantly enhances the expression of stemness markers (PEDF, HES1) in LESCs, while conditioned media from niche cells (mesenchymal stromal cells and melanocytes) further supports this undifferentiated phenotype. This underscores the necessity of biomimetic strategies in maintaining stem cell potency for clinical applications (Aghazadeh et al.).

Looking beyond human models, comparative biology offers unique perspectives on regeneration. Boldyreva et al. investigate the adipose-derived stem cells (ADSCs) of *Acomys cahirinus* (the spiny mouse), a mammal capable of scar-free skin and tissue regeneration. They report a fascinating trade-off: *Acomys* ADSCs exhibit a distinct osteogenic shift and reduced adipogenic potential compared to *Mus musculus*, alongside suppressed lipolysis in their adipose tissue. This suggests that the metabolic and differentiation flexibility of stem cells is a pivotal evolutionary adaptation in organisms with high regenerative capacity, offering new targets for bio-inspired regenerative strategies (Boldyreva et al.).

## Cell-free therapies: the rise of extracellular vesicles

While cell transplantation remains a cornerstone of therapy, the field is increasingly pivoting towards cell-free approaches to circumvent Research Topic of immunogenicity and stability (Phinney and Pittenger, 2017). Mesenchymal Stem Cell-derived Extracellular Vesicles (MSC-EVs) have emerged as powerful mediators of regeneration. Zhang et al. comprehensively review these “Tiny Giants,” detailing their ability to cross biological barriers and deliver bioactive cargos that modulate inflammation and promote angiogenesis across diverse tissues, from bone to brain (Zhang et al.).

Building on this, Chen et al. focus on the specific application of exosomal secretomes in Anterior Cruciate Ligament (ACL) reconstruction. They elucidate how stem cell-elicited microenvironmental reprogramming—specifically through the regulation of macrophage polarization and vascular remodeling—can overcome the poor intrinsic healing capacity of the ACL. Their review highlights the potential of engineered exosomes to prevent scar formation and enhance tendon-bone integration, a critical clinical hurdle (Chen et al.).

## Targeting the microenvironment: inflammation, clearance, and apop*tosis*

Regeneration is not merely about adding new cells but also about resolving the pathological microenvironment that inhibits repair (Forbes and Rosenthal, 2014).Several contributions to this Research Topic address the critical role of immune modulation and cell death pathways.

Duan et al. present a compelling review on “Efferocy*tosis*”—the phagocytic clearance of apoptotic cells—describing it as a “Death as Rebirth” mechanism. They articulate how efficient efferocy*tosis* drives the switch from inflammation to tissue repair. The authors discuss innovative delivery platforms, including nanomaterials and hydrogels, designed to restore defective efferocy*tosis* in conditions ranging from atherosclerosis to cancer, thereby re-establishing tissue homeostasis (Duan et al.).

In the context of chronic pathologies, Xu et al. propose a novel paradigm for treating Chronic Subdural Hematoma (CSDH). Moving away from the view of CSDH as a static fluid Research Topic, they characterize it as a dynamic, *inflammatory* microenvironment driven by leaky neovascularization and aberrant fibrosis. Their review advocates for regenerative interventions that target these upstream microenvironmental drivers rather than simple surgical evacuation (Xu et al.).

Similarly, Chen et al. tackle the molecular roots of Intervertebral Disc Degeneration (IDD). They focus on the regulation of Nucleus Pulposus Cell (NPC) apop*tosis* by microRNAs. By summarizing anti-apoptotic miRNA targets and reviewing advanced delivery strategies—such as NPC-targeting nanoparticles and responsive hydrogels—they provide a roadmap for gene therapies aimed at arresting the cascade of disc degeneration (Chen et al.).

## Translational frontiers in complex diseases

Finally, the Research Topic extends into systemic and neurodegenerative diseases, where stem cell therapies face their rigorous tests. Shi et al. review the progress of stem cells (MSCs, iPSCs, EPCs) in treating Atherosclerosis. They emphasize the multi-modal mechanism of these cells, which not only repair vascular endothelium but also stabilize plaques through immunomodulation and lipid *metabolism* regulation, offering a potential disease-modifying therapy for cardiovascular conditions (Shi et al.).

In the realm of neurodegeneration, He et al. explore the promise of stem cell therapy for Alzheimer’s disease. Their review synthesizes evidence on how stem cells can replace lost cholinergic neurons, secrete neurotrophic factors, and enhance amyloid-beta clearance. While acknowledging challenges like the blood-brain barrier, they highlight the potential of combining stem cells with gene editing and organoid models to pave the way for clinical translation (He et al.).

## Conclusion

Collectively, the articles in this Research Topic illustrate that the future of tissue regeneration lies in precision. Whether through the specific molecular tuning of the stem cell niche, the engineering of exosomal cargos, or the targeted modulation of inflammatory and apoptotic pathways, the field is moving towards therapies that do not just patch defects but reprogram the biological machinery of repair. We hope this Research Topic inspires further research into these cellular mechanisms, ultimately translating novel insights into tangible clinical benefits.
